# The impacts of climate change, energy policy and traditional ecological practices on future firewood availability for Diné (Navajo) People

**DOI:** 10.1098/rstb.2022.0394

**Published:** 2023-11-06

**Authors:** Kate Magargal, Kurt Wilson, Shaniah Chee, Michael J. Campbell, Vanessa Bailey, Philip E. Dennison, William R. L. Anderegg, Adrienne Cachelin, Simon Brewer, Brian Frank Codding

**Affiliations:** ^1^ Environmental and Sustainability Studies and SPARC Environmental Justice Lab, University of Utah, Salt Lake City, UT 84112, USA; ^2^ Department of Geography, University of Utah, Salt Lake City, UT 84112, USA; ^3^ Department of Biology, University of Utah, Salt Lake City, UT 84112, USA; ^4^ Department of Anthropology, University of Utah, Salt Lake City, UT 84112, USA; ^5^ Department of Admissions, Diné College, Tsaile, AZ, USA

**Keywords:** energy sovereignty, energy equity, indigenous ecological knowledge, socio-environmental systems, participatory agent-based modelling, environmental justice

## Abstract

Local-scale human–environment relationships are fundamental to energy sovereignty, and in many contexts, Indigenous ecological knowledge (IEK) is integral to such relationships. For example, Tribal leaders in southwestern USA identify firewood harvested from local woodlands as vital. For Diné people, firewood is central to cultural and physical survival and offers a reliable fuel for energy embedded in local ecological systems. However, there are two acute problems: first, climate change-induced drought will diminish local sources of firewood; second, policies aimed at reducing reliance on greenhouse-gas-emitting energy sources may limit alternatives like coal for home use, thereby increasing firewood demand to unsustainable levels. We develop an agent-based model trained with ecological and community-generated ethnographic data to assess the future of firewood availability under varying climate, demand and IEK scenarios. We find that the long-term sustainability of Indigenous firewood harvesting is maximized under low-emissions and low-to-moderate demand scenarios when harvesters adhere to IEK guidance. Results show how Indigenous ecological practices and resulting ecological legacies maintain resilient socio-environmental systems. Insights offered focus on creating energy equity for Indigenous people and broad lessons about how Indigenous knowledge is integral for adapting to climate change.

This article is part of the theme issue ‘Climate change adaptation needs a science of culture’.

## 1. Introduction

The world is undertaking a global energy transition in its attempt to stem emissions from the burning of fossil fuels [1,2]. Just as people who face the direst consequences of anthropogenic climate change contributed least to its cause [3–7], so are marginalized communities at most risk of accruing more costs than benefits during this energy transition [8–10]. However, these same communities often hold place- and culturally appropriate expertise relevant to adapting to ecological change due to shifting climate conditions.

Indigenous communities throughout the world are at higher risk of environmental hazards from climate change compared with other demographics due to a suite of vulnerability factors, including political marginalization, economic and health inequality, and diminishment of access to traditional lands that constrain possibilities for making a living [11,12]. At the same time, many such communities' long tenure in place mean they hold intergenerational expertise about how to understand and adapt to local environmental change and continuously engage in adaptive processes that derive from that expertise [13–15]. Knowledge and practice developed over generations of interactions tethered to specific places and environments is integral to cultural adaptations to climate change since such knowledge represents adaptive strategies tested against past times of change. Such knowledge is often referred to as Indigenous Ecological Knowledge (IEK) [16–20]. This paper explores the conditions that either facilitate or prohibit communities from successfully applying community-held expertise, such as IEK, to adapt to climate change.

The dynamics between national energy policy, trends in energy economics, and issues around Indigenous land tenure and Tribal sovereignty converge to create a landscape where IEK practitioners may be helped or hindered. On one hand, sovereignty over traditional lands and land-based practices allow Indigenous communities to maintain access to traditional resources [21–26]. Around the world, movements to return traditional lands to the jurisdiction of Indigenous governance or to co-manage such lands has been on the rise in recent decades [27,28]. On the other hand, national energy policies and economics tend to focus on industrialized energy sources, creating obstacles to adaptive strategies that incorporate traditional energy sources. Energy is a key resource often exported from Native lands while community members themselves struggle to meet energy needs due to a combination of historically forced reliance on—and disenfranchisement from—industrialized energy systems, particularly (but not limited to) fossil fuels [29–31]. Energy is intertwined with issues of access, traditional cultural practice and knowledge, and continuing fights to achieve energy sovereignty [30–32]. Community-based adaptations are diverse, and often include weaving the application of new technologies with long-held knowledge and practice. Much discussion focuses on the development and deployment of new technologies, such as those that generate electricity from solar panels. This discourse often overlooks the enduring role of IEK as an element of climate adaptation, and mitigation. Firewood and charcoal are an important source of traditional energy that roughly a third of the world relies on [33]. Importantly, firewood is fundamental to IEK-based practices and Indigenous self-determination [32,34,35]. And, critically, the woodlands upon which individuals rely are threatened by climate change [36,37].

Our work on the northern part of Navajo Nation and surrounding lands exemplifies these dynamics. Here we examine convergent influences of climate change and energy policy on Diné wood-hauling practices. We begin by describing the cultural and ecological context of wood hauling in northern Diné communities. We explore local expertise, collected through ethnographic methods and previous analysis, about the influence on IEK exerted by exogenous factors of climate change and shifting trends in the energy industry. Finally, we present the results of an agent-based modelling (ABM) experiment, trained with the expertise contributed by Diné Tribal members, current environmental conditions, and projected future climate scenarios to explore the conditions where IEK facilitates adaption to changing conditions by promoting sustainable firewood harvesting and healthy woodland ecosystems into the future.

## 2. Geographical, cultural and climate context of wood hauling

Diné ‘woodhaulers’—the local term for folks who gather their own firewood from regional woodlands and forests—are situated in a diverse and dynamic ecological, cultural and economic context. Prior to the arrival of European explorers, and later Euro-American colonists, Diné people moved seasonally among a variety of ecological contexts ranging across arid canyons and plateaus to alpine forests, alongside numerous neighbouring Indigenous cultures. These traditional lands include large portions within the current political boundaries of Utah, Arizona, Colorado and New Mexico within the USA. Successive waves of Euro-American expansion into the region spurred ever-increasing limits on access to traditional lands for Indigenous people, enforced by numerous colonial practices. These practices include many codified by varying levels of non-Indigenous governments (for a more expansive treatment of this history, see [32]). Although IEK continues to evolve, the ability of IEK practitioners to fully exercise this knowledge is constrained by a suite of structurally discriminatory social geographies, policies and practices. For example, the reservation system distances people from traditional lands, and government systems (such as state and federal land management) frequently further disenfranchises people. The legal requirement for firewood permits is one example [32].

Firewood (*chizh* in Diné) is an important aspect of the energy economy of Diné, providing a medium for participation in local ecology—mediated through IEK—as well as a primary source of household energy. Two types of trees provide most of the firewood harvested; pinyon (called *chá'ol* in Diné, *Pinus edulis*) and juniper (frequently called cedar in English, or *gád* in Diné, *Juniperus* spp). In many Diné households, particularly in the northern and western parts of Navajo Nation, firewood is supplemented with coal to fully meet energy needs. This dynamic between the burning of coal and firewood is complex: many Diné acknowledge a reluctance to burn coal at home because of the well-known negative environmental and health effects [38], but at the same time, it is often the only accessible option, in combination with firewood, to meet household needs. Many rural Diné households are not connected to the electrical grid [39], and meeting home energy needs via household solar is frequently not sufficient to maintain the high-energy draw of heating. A coal mining complex on Black Mesa, Arizona, including what were known as the Black Mesa and Kayenta Mines, provided public coal fields beginning in the 1970s. Diné, along with neighbouring Hopi households, from the region gathered coal from these public piles and purchased coal by the truckload for relatively low cost (approx. 70–100$/truckload, depending on the weight). This coal provided cheap and reliable fuel to burn in household appliances to supplement firewood. The last mining operation on Black Mesa closed in late 2019 and the public piles were shuttered. Although ongoing efforts seek to add electrical capacity to Diné households by connecting them to existing electrical grids or donating off-grid household photovoltaic systems, these efforts fall far short of supplanting the need for burning solid fuels. Gridded electricity is still absent from large swaths of Navajo Nation, and participants in this study reported frequent outages even where connections exist. Household photovoltaic solar systems are often promoted as an energy solution; however, study participants report that low-energy generating capacity of many such systems means that high-draw appliances such as those involved with heating cannot be supported by current installations. Also, although numerous non-profits match donations of off-grid solar power technology to households in need, a lack of capacity for tending to ongoing maintenance results in short lifespans for these systems. Many of these investments, while welcomed and often desired by Diné community members, are also frequently not community-generated. Study participants cite some hope for adopting solar technologies, but nearly all believe that firewood is the most reliable and culturally appropriate way to ensure homes are kept warm and ceremonies are able to continue. Thus, under current conditions, without coal readily and cheaply available, the demand for firewood increases. Firewood harvest practices that adhere to IEK involve avoiding the harvest of green wood and provide for sustainable resource use; however, the increasing pressure on woodland resources, coupled with the climate-driven decrease in those resources over time, may limit the ability to practice IEK in the absence of viable alternatives such as accessing additional woodlands, decreasing energy needs or supplementing firewood with other energy sources.

**Figure 1.**
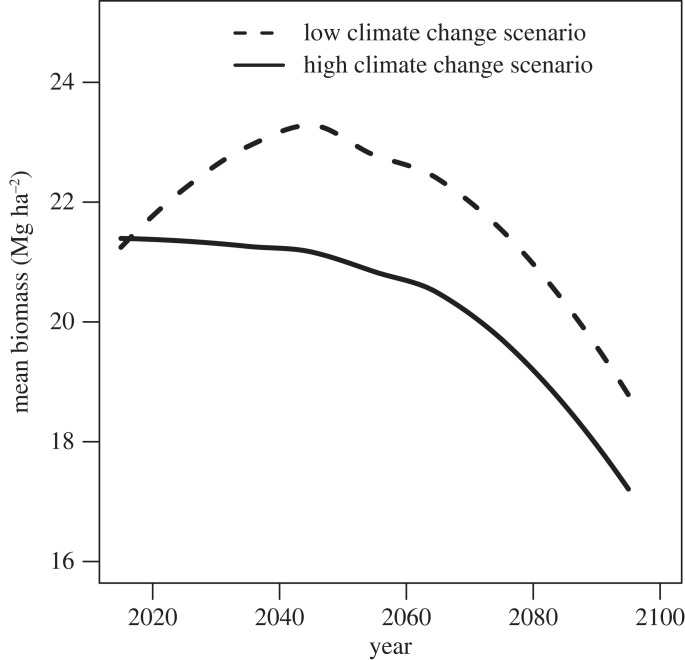
Biomass—the total amount of plant material—in pinyon-juniper woodlands will decrease in the coming decades [41,47]. The dashed line shows what is likely under a low climate change future scenario if human society drastically reduces global CO_2_ emissions. The black line shows what is likely under a high climate change future scenario if global CO_2_ emissions continue at current rates. Further detail about how these scenarios are derived is given in electronic supplementary material S2, §3.1.1.

In addition to systemic political and social restrictions to accessing firewood, climate change is also impacting regional woodlands in ways that will reduce future potential firewood harvest. Drought has already instigated a series of arboreal mortality events in the pinyon-juniper woodlands of southeast Utah in the last two decades [40] and such conditions are expected to continue and intensify in this century [41–47]. Our prior work shows that live biomass in pinyon-juniper woodlands in San Juan County, Utah, are expected to decrease between 13 and 21% by the end of this century, including in places important to Diné wood haulers (figure 1, [41,47]). Increasing drought conditions also impact Diné communities in myriad additional ways. Many elders note decreasing snowfall, increasing dust and wind storms, decreasing plant and animal diversity, and decreasing availability of surface water across Navajo Nation [48].

Shifts in temperature and precipitation patterns are predicted to bring substantial consequences for the world's arboreal ecosystems, especially arid forests and woodlands [49]. Woody biomass from the pinyon-juniper woodlands is distributed heterogeneously throughout the study region. While the distribution of biomass influences where people harvest firewood, other social, economic and cultural factors, including the application of IEK, also dictate where firewood is harvested. We consider these factors as part of a broad human-ecological system which, in this case, forms the basis of decisions people make about when and where to harvest firewood.

We examine the potential of energy-oriented IEK to buffer Indigenous communities against climate-induced threats to energy security and sovereignty. Using a mixed methods approach, we

(i) detail a system of interactions between people and woodlands mediated through IEK, including how this system is likely to be impacted by climate change,
(ii) demonstrate how IEK, when free of constraints in application, creates adaptive outcomes for human-ecological systems, and
(iii) explore how changes in demand caused by broader energy transitions impact the ability to adhere to IEK and thus, practice and develop locally appropriate climate adaptation practices.

## 3. Methods

**Figure 2.**
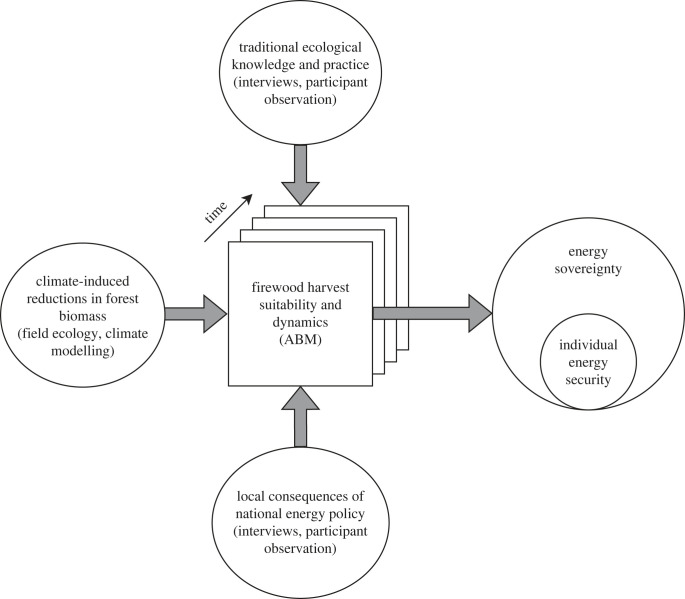
We explore how IEK and energy transitions influence energy security and sovereignty through a systems model summarized in this figure. The distribution of harvestable wood on the landscape influences where people harvest firewood. Climate change will lead to decreased tree growth in woodlands where firewood is gathered over the next century, causing changes to the distribution of harvest. IEK also influences the distribution of firewood harvest through informing the condition of trees that are appropriate for harvest. The local consequences of national energy policy and economics influence the distribution and intensity of firewood harvest by influencing access to alternative energy sources such as coal. The ability of individuals to access firewood through harvest influences individual energy security. Finally, the dynamics of this system influence energy sovereignty alongside other factors beyond this framework.

This study was initiated by the native-lead non-profit organization, Utah Diné Bikéyah (UDB), who was informed about the importance of firewood to traditional lifeways of the region's Tribes through a series of elder interviews begun in 2009. Academic researchers and UDB formed a collaborative team that included IEK experts, wood haulers, geographers, biologists, atmospheric scientists, environmental justice scholars and anthropologists. Representatives from these teams comprise the authorship of this paper. Community members whose lived experiences and IEK involve wood hauling guided the development of the methods and provided iterative feedback, facilitating a community-based approach to the conventional ethnographic methods described below. Our methods and results sections first report on our community-based ethnographic data collection, followed by the ABM experiment. A schematic of how data collected interfaces with the ABM and conceptual interpretations is given in figure 2. Essentially, we combine ecological and ethnographic models, trained by empirical observations, to explore how adherence to IEK might impact future outcomes for Diné communities and pinyon-juniper woodlands.

### (a) Compiling expertise of Diné wood haulers

We apply mixed methods to quantify the shifting access to energy for Diné over time and contextualize these dynamics in the cultural, historical, economic and ecological legacy in which they occur. Through this combined approach, we describe how IEK operates in ways that provide adaptive flexibility [50], but which is also constrained by ongoing exogenous factors such as the combined impacts of climate change and shifts in industrial energy economy.

Tribal members communicated their knowledge and experience about firewood harvest and use through 9 interviews, 120 survey responses and 54 focal follows. Both the University of Utah Institutional Review Board (IRB no. 00090654) and the Navajo Nation Human Research Review board reviewed the study, including sample questionnaires, and exempted the study from specific oversight. A cultural resources investigation permit was approved by the Navajo Nation Heritage and Historic Preservation Department (permit no. C18024-E). The Navajo Utah Commission also passed a resolution in support of this research (resolution no. NUCFEB-714-18). Surveys asked respondents to quantify wood harvest and uses by species and to map wood harvest areas in relation to home sites. Survey information was digitized from paper forms into excel spreadsheets and GIS software. Semi-structured interviews were conducted to explore ideas and experiences related to wood harvest, and were recorded and transcribed then coded and compiled using NVivo [51]. Focal follows (where researchers joined wood haulers as they harvested wood) allowed for the collection of directly observed information and *in situ* informal interviews. This information included details of IEK and its application, species selection and quantified aspects of wood harvesting such as travel times, the amount of wood per load, and more details about species selection. These data inform both qualitative and quantitative aspects of firewood harvest. The primary findings from this work involve characterizing the main expressions of IEK in the practice of firewood harvest and community experiences on the impact of the closing coal mine on firewood demand. Details on data flows used in the model are given in the electronic supplementary material S2, §5.

### (b) Agent-based modelling of firewood harvest decisions given changes in exogenous variables

To understand complex emergent patterns in how changes in the supply of woody biomass, increasing levels of demand driven by energy transitions, and adherence to IEK impact the pinyon-juniper woodlands and household energy security for wood haulers, we employ an agent-based modelling (ABM) approach [52–54]. We felt this the most appropriate modelling tool given the ability of ABMs to: (i) incorporate spatial variation in vegetation distributions, which is necessary for understanding emergent outcomes for species selection and travel costs; (ii) track emergent outcomes for both woodland and human agent populations, including spatial dimensions; and (iii) be easily adaptable to future analyses which add dynamics that we or other scholars might wish to explore. Using Netlogo [55], we constructed the ABM to enable interactions between tree populations and firewood harvesting dynamics where we examine the effect of a range of levels of adherence to IEK as demand and supply varies. Electronic supplementary material S1 provides a Netlogo file and electronic supplementary material S2 provides an Overview, Design Concepts and Details summary (ODD, following the standard set by Grimm *et al*. [56–58]). Key independent variables in the model are IEK (valuation of live wood), demand (impact of addition or loss of alternate energy streams) and climate-driven supply. Climate-driven supply is estimated as the impact on live biomass resulting from three possible CO_2_ emission scenarios: (i) stable supply, (ii) supply diminished to an extent possible in a low climate change scenario (the dashed line in figure 1), and (iii) supply diminished to the extent possible in a high climate change scenario (the solid line in figure 1). Section 3.1 in electronic supplementary material S2 offers greater detail of how climate change induced decreases in biomass are operationalized in the ABM.

The model is trained with empirical observations and community-reported information including which species are the primary focus of firewood harvest and the average amount of wood needed per year (reported below and in [32]). Validation of the model was conducted by observing the minimum time intervals required for each state to reach equilibrium and choosing those parameters as reasonable for model runs. Additional validation measures involved perturbing variables beyond designed ranges and observing whether model outcomes violated theoretical expectations. Further detail about model validation are given in electronic supplementary material S2. Owing to stochasticity in woodland initiation, tree recruitment, tree death and tree growth, 100 model runs are conducted for each combination of the independent variables: (i) level of adherence to IEK, (ii) variation in demand for firewood, and (iii) variation in firewood supply. The experiment therefore consists of 270 model runs. Outputs from the model runs were compiled into comma-delimited values and imported into *R* for analysis (electronic supplementary material S3 contains the data table; [59]). The response variables examined are: (i) the proportion of foragers in the model meeting their firewood demand, (ii) the distance travelled by foragers as they access firewood (a proxy for cost of harvest), (iii) the total biomass and mean live tree age of pinyon, and (iv) the total biomass and live tree age of juniper. Output variables are evaluated using standard response plots. For more detailed descriptions of the model, see electronic supplementary material 1:4.

## 4. Results

### (a) Ethnographic findings

Firewood-related IEK is embedded in a suite of human-ecological connections to the woodlands where wood is harvested. One aspect of IEK relates to selecting wood appropriate for burning as firewood, as exemplified in one interviewee's statement, ‘we don't bother the live trees’. All practitioners interviewed, surveyed, or who participated in focal follows reported that firewood should only be taken from dead wood, ideally from recently deceased trees which are frequently recently fallen, but sometimes still standing. The collection of live wood is acceptable only for specific construction and ceremonial purposes and is taboo when the use is for firewood. Despite this taboo, some Diné wood haulers shared that they have observed the harvest of live trees for firewood. Most of these observations were reported to occur on Tribal lands in regions where woodland cover is extremely limited. We interpret this contradiction as stemming from the exact convergence of factors we seek to explore—limited supply combined with high demand. Travel costs to woodlands where firewood is available are frequently high (up to 120 km, [32]), and to harvest a truckload of firewood requires a substantial investment in time and resources. The costs of harvesting firewood are multiplied by the number of trips required to meet each year's needs, reported by survey respondents to be seven truckloads on average. In areas where people have little alternative for heat, and little opportunity to pay the costs associated with alternatives, people are more likely to abandon IEK out of necessity.

In early 2019, wood haulers alerted us to the planned closure of the public coal piles at the Kayenta mine, scheduled and eventually executed in autumn of 2019. Study participants expressed deep concern about the implication of this closure for people's ability to meet their energy needs as well as the potential impact of the likelihood that many would make up the resulting shortfall by increasing their demand for firewood or simply be cold. One interviewee notes, ‘[…] how's everybody gonna stay warm during the winter now since there's no more [coal]? […] all we're using is just the wood […], and it's okay but like, it doesn't stay on and stay warm for quite a long time.’ The interviewee refers to the fact that many people rely on coal not only to reduce their need for firewood, but to also reduce the amount of fire tending required when coal is available. Many use longer-burning coal in home heating appliances overnight to ensure consistent heating while asleep.

We operationalized two aspects of this community expertise into our ABM: (i) that full adherence to IEK involves the harvest only of dead wood for firewood and (ii) that reducing access to coal for home heating would increase demand for firewood.

### (b) Agent-based model results

**Figure 3.**
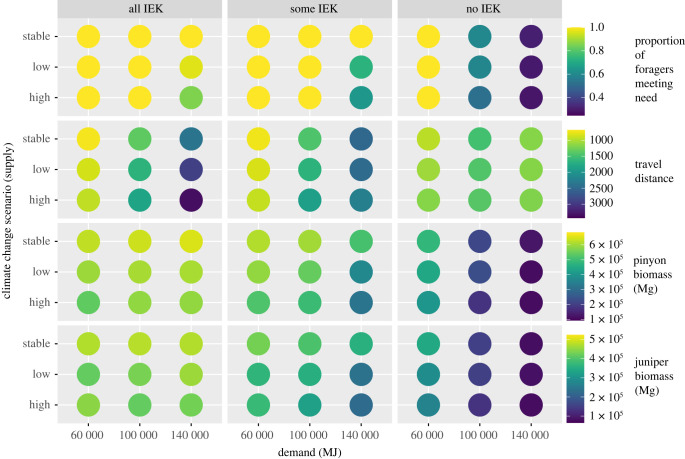
Patterning in the mean annual response variables given different levels of IEK adherence and variation in supply and demand. The demand for firewood varies on the *x*-axis across the three IEK scenarios. Supply varies on the *y*-axis as a function of emissions scenario, where ‘stable’ refers to a scenario without climate change (the highest supply level), ‘low’ refers to a low-emissions scenario where supply is only reduced slightly, and ‘high’ refers to a high-emissions scenario where supply is reduced substantially. Coloured dots in each parameter space represent measures for the outcome variable shown to the right of each row.

The results of each ABM scenario are summarized in figure 3. Values show the yearly mean over all runs in each model scenario. For each parameter combination, we are interested in identifying outcomes where (i) harvesters are able to meet their needs, while (ii) paying the lowest travel costs, and where (iii) woodland biomass persists into the future. Below we unpack these outcomes relative to the main parameter combinations relating IEK, climate-driven supply and household demand.

#### (i) Meeting household need

The proportion of foragers meeting their household need is maximized under scenarios with greater adherence to IEK, when climate is stable or experiencing low-level climate change, and when demand is low. Importantly, strict adherence to IEK practices results in all or nearly all households meeting demand under all but the most pessimistic climate and demand scenario combinations. Additionally, nearly all households can meet their need under low community demand scenarios even when emissions are high and IEK practices are not followed. This suggests that access to alternative energy sources can successfully mitigate failures to curb global emissions and a lack of adherence to IEK.

#### (ii) Acquisition costs

Long-term travel costs are minimized under scenarios where individuals adhere strictly to IEK, climate is stable or experiencing low-level climate change, and demand is low. The highest average travel costs are incurred under strict IEK, the high climate change scenario and high demand. This result emerges due to the climate- and demand-driven constraints on the availability of dead wood, which forces individuals following IEK to travel further, indicating potential conflict between preferred harvesting strategies and economic self-interest, and thus a scenario where IEK is unlikely to be able to be maintained. Interestingly, travel costs are moderate to high under all scenarios when harvesters do not adhere to IEK, because harvesting live wood results in local deforestation radiating out from the central place, thereby requiring longer, and longer travel times through model runs. Keeping demand lower appears to have the greatest effect on keeping travel times low.

#### (iii) Woodland biomass

Long-term pinyon and juniper biomass is greatest under scenarios where agents strictly adhere to IEK, and climate is stable or experiencing low-level climate change. This suggests that woodland health benefits most from IEK practices. Biomass is lowest under scenarios where wood haulers remove live wood, especially when demand and climate change are at high levels. Interestingly, the greatest pinyon biomass emerges under high IEK, stable climate and high-demand scenarios. The greatest juniper biomass similarly emerges under high IEK, and high demand, but under the low climate change scenario. This suggests a complex dynamic where IEK can promote woodland health even under pessimistic future scenarios.

## 5. Discussion

### (a) Implications for future socio-environmental systems in pinyon-juniper woodlands

Results indicate that both woodlands and wood haulers do better over the long term if harvesting strategies follow IEK, especially if global greenhouse gas emissions and local demand for wood are lowered. Importantly, this socio-environmental system appears resilient even under the high climate change scenario, at least within the projections of biomass considered here (and long-term biomass projections are generally quite uncertain in the USA, [47]), as long as harvesting incorporates IEK to focus on dead wood and demand is kept to current or lowered levels. In this local context, keeping demand low may necessitate a suite of solutions that should include improvements to home structures to increase energy efficiency, planning for an increase in access to both short- and long-term alternative household energy sources when retiring conventional sources such as coal, and facilitating the practice and transmission of IEK. These dynamics are complex and elaborated on usefully for Navajo Nation elsewhere (e.g. [31,32,44,60]). The central point of this paper is that IEK is a necessary component of an actively adaptive human–environment system in pinyon-juniper woodlands.

Additionally, as long as demand is kept to current or lower levels, and wood haulers mostly adhere to IEK-informed strategies, then individuals will experience lower average long-term acquisition costs. This is because strategies that ignore IEK and take standing live wood deplete local stands, resulting in longer average travel times over the long term. This finding suggests that wood haulers benefit in the long run by following IEK, despite the short-term costs of having to travel further. The woodlands themselves benefit as well since biomass persists best with IEK, suggesting this socio-environmental system may result from long-term coevolutionary dynamics common in other regions [61–64].

### (b) Indigenous ecological knowledge as adaptive practice that supports energy security and sovereignty

As discussed above, Diné wood hauling today exists in a complex economic and political landscape that marginalizes Diné communities from ‘industrialized’ energy sources. However, it would be inaccurate to cast this marginalization solely in a framework of ‘energy poverty’ [65] because of the strong cultural values associated with firewood. These cultural values range across important aspects of Diné identity (without firewood, we would not be Diné) to concerns about the environmental impacts of industrialized energy sources [66]. IEK is embedded in these cultural values and sustains a system wherein Diné cultural identity, including firewood harvest and use, can be sustained. Because of how IEK sustains characteristics of this human-ecological system, Diné wood haulers can depend on access to firewood even as access to other energy sources for household use are unreliable. Up till now, the reliability of firewood supports energy security by allowing individuals to heat their homes in culturally relevant ways. Firewood buffers the risk of energy shortfalls when electricity or other resources are economically or institutionally out of reach, due to unaffordability and disenfranchisement from systems of industrialized energy infrastructure, respectively. When sovereignty is defined as being driven by self-determination, culturally appropriate energy sources such as firewood, sustained through IEK-driven practice is integral to sovereignty. Thus, when exogenous forces push people to abandon IEK out of necessity for meeting short-term needs, both energy security and sovereignty are at risk.

Exogenous factors can help to relieve the pressures that may push people away from IEK. Such factors can include those directly related to global efforts to decarbonize while supporting marginalized communities, such as the Interagency Working Group on Coal & Power Plant Communities & Economic Revitalization [67] which identifies the very communities represented by this work as eligible for investment in new economic opportunities in the wake of departing fossil-fuel-based economic engines. The present work suggests that such an intervention, a climate adaptation itself on the national scale, will succeed best if it is applied by those who know how to weave it with ongoing cultural processes such as IEK. These successes would likely be measured in outcomes far beyond the economic and ecological ones mostly mentioned here. The details we explore in this paper capture only some dimensions of how Diné households respond to these continued and increasing pressures on their energy and cultural systems. Weaving IEK as a form of cultural identity integral to the future would shift the heavy psychological and emotional burden carried by Diné—and many other Native communities—to an empowering form of both continued cultural life and a significant contribution to the global community as we grapple with how to confront climate change. Such culturally informed practices are not limited to the harvest of firewood guided by IEK, but extend to how firewood is shared throughout communities, supported by the relationships tended by ceremonies, hauling wood together and numerous other important activities.

In addition to the ways in which fostering IEK promotes desirable outcomes for human communities, it also may mitigate impacts of climate change on arboreal ecosystems. The ability of the world's forests and woodlands to sustain their function as carbon sinks while undergoing climate-induced change is currently unclear [41]. The role of local human–woodland interactions continues to be debated, but frequently focuses on land-use change [68,69] rather than the importance of maintaining human-ecological legacies [70]. Our findings support the notion that IEK-informed woodland management strategies will not only yield the most desirable humanitarian outcomes, but also sustain important characteristics of a resilient woodland ecosystem. Decision-makers at all levels should consider how the consequences of larger trends may produce conditions that reduce the likelihood of IEK practitioners to succeed in maintaining necessary elements of human-ecological legacies.

## Data Availability

Data are available from the Dryad Digital Repository: https://doi.org/10.5061/dryad.xwdbrv1k8 [71]. Data and code are provided in the electronic supplementary material [72].
